# Treatment of Ingrown Toe-Nail

**Published:** 1879-09

**Authors:** 


### TREATMENT OF INGROWN TOE-NAIL.

DR. JAMES MCEVOY says, in the *Louisville Medical News*, that he has found the following simple plan effective:

A small wedge-shaped piece of sponge tent is cut and inserted under the ingrown part of the nail as far as possible. The size of the sponge is increased at each insertion, which is generally every third day. If there is a foetid discharge the sponge is dipped in carbolized oil before using.

In the *Chicago Medical Journal and Examiner*, Dr. Andrews gives the following operation as that of a chiropodist named Willard: He neither extracts the nail nor slices off the overlapping flesh, but cuts out a narrow elipse of tissue near the nail and parallel to its border, claiming that the border itself where it rests against the edge of the nail has its special structure adapted to its location, and ought not to be sacrificed. The removal of the strip of flesh being accomplished, he brings the edges of the wound together with fine sutures, thus drawing the border away from the nail and effecting a cure.

